# Functional Analysis of *MaWRKY24* in Transcriptional Activation of Autophagy-Related Gene *8f/g* and Plant Disease Susceptibility to Soil-Borne *Fusarium oxysporum* f. sp. *cubense*

**DOI:** 10.3390/pathogens8040264

**Published:** 2019-11-25

**Authors:** Guoyin Liu, Hongqiu Zeng, Xiang Li, Yunxie Wei, Haitao Shi

**Affiliations:** Hainan Key Laboratory for Sustainable Utilization of Tropical Bioresources, College of Tropical Crops, College of Forestry, Hainan University, Haikou 570228, China; liuguoyin@hainanu.edu.cn (G.L.); 17090102110001@hainanu.edu.cn (H.Z.); 20177407320071@hainanu.edu.cn (X.L.); 994016@hainanu.edu.cn (Y.W.)

**Keywords:** autophagy, banana (*Musa acuminata*), soil-borne *Fusarium oxysporum* f. sp. *cubense*, transcription factor, WRKY

## Abstract

WRKYs play important roles in plant development and stress responses. Although *MaWRKYs* have been comprehensively identified in the banana (*Musa acuminata*), their in vivo roles and direct targets remain elusive. In this study, a transcript profile analysis indicated the common regulation of *MaWRKYs* transcripts in response to fungal pathogen *Fusarium oxysporum* f. sp. *cubense* (*Foc*). Among these *MaWRKYs*, *MaWRKY24* was chosen for further analysis due to its higher expression in response to *Foc*. The specific nucleus subcellular location and transcription activated activity on W-box indicated that *MaWRKY24* was a transcription factor. The correlation analysis of gene expression indicated that *MaWRKYs* were closely related to autophagy-associated genes (*MaATG8s*). Further analysis showed that *MaWRKY24* directly regulated the transcriptional level of *MaATG8f/g* through binding to W-box in their promoters, as evidenced by quantitative real-time Polymerase Chain Reaction (PCR), dual luciferase assay, and electrophoretic mobility shift assay. In addition, overexpression of *MaWRKY24* and *MaATG8f/g* resulted in disease susceptibility to *Foc*, which might be related to the activation of autophagic activity. This study highlights the positive regulation of *MaWRKY24* in transcriptional activation of autophagy-related gene *8f/g* in the banana and their common roles in disease susceptibility to soil-borne *Foc*, indicating the effects of *MaWRKY24* on autophagy and disease susceptibility.

## 1. Introduction

In recent years, the widespread soil-borne fungus *Fusarium oxysporum* (*Fo*) has caused vascular wilt disease and serious yield loss in crops [1,2]. Fusarium wilt of the banana (Panama disease) caused by soil-borne *Fusarium oxysporum* f. sp. *cubense* (*Foc*) is one of the most destructive [3,4,5,6]. What is worse, the soil-borne disease spreads rapidly, and no sustainable control method has been developed so far, thus posing a great threat to banana production all over the world [3,6,7,8]. Based on different banana hosts, at least three strains of *Foc* have been identified: *Foc* strain 1 (*Foc* 1), *Foc* strain 2 (*Foc* 2), and *Foc* strain 4 (*Foc* 4) [4,6]. The initial symptom of banana wilt is blister damage on the leaves, followed by progressive leaf yellowing from the lower leaves to the upper leaves in response to *Foc* infection [8]. Unfortunately, although some differently expressed genes have been identified in response to *Foc* infection [2], their roles remain unclear in vivo. Therefore, functional analysis of *Foc*-regulated genes might be helpful to better understand the molecular processes related to disease resistance and develop a disease-resistant variety through genetic breeding.

WRKY transcription factors are defined based on their DNA binding domain with WRKY amino acid sequences of 60 residues in the N-terminus. Moreover, they have an atypical zinc finger structure of Cx4-5Cx22-23HxH or Cx7Cx23HxC in the C-terminus [9,10,11]. WRKY transcription factors are widely involved in plant development and stress responses. On the one hand, WRKYs can be served as activators or repressors to modulate various plant processes [12,13]. On the other hand, WRKYs can interact with other proteins, such as mitogen-activated protein (MAP) kinase (MAPK) [10,14,15,16], MAP kinase kinase kinase (MEKK) [10,16,17], calmodulin [10], and histone deacetylases (HDAs) [10]. For example, the phosphorylation of *WRKY7/8/9/11* by MAPK accelerates WRKY-dependent respiratory burst oxidase homolog (RBOHB) expression via binding to the cognate W-box in the promoter, resulting in ROS burst in *Nicotiana benthamiana* [18]. Interestingly, *AtWRKY53* is phosphorylated by MEKK1; this may increase the binding of *AtWRKY53* to its own promoter and regulate senescence [10].

WRKYs have been widely investigated in plants. For example, *CsWRKY31* (*Camellia Sinensis*, Cs) and *CsWRKY48* can inhibit the transcriptions of biosynthesis-related genes leucocyanidin reductase (*LAR*), dihydroflavonol-4-reductase (*DFR*), and caffeoyl-CoA 3-O-methyltransferase (*CCoAOMT*) through binding to the W-box elements, served as negative regulators of O-methylated catechin biosynthesis [12]. WRKYs can also regulate leaf senescence by interacting with DELLA protein repressor of ga1-3-LIKE1 (RGL1) in the gibberellin signaling pathway [19], the epithio specifying senescence regulator (ESR/ESP) in salicylic acid (SA), and the jasmonic signaling pathway [20], respectively. Furthermore, the ectopic expression of *VqWRKY52* (*Vitis quinquangularis*, Vq) in *Arabidopsis thaliana* enhances the resistance to powdery mildew and *Pseudomonas syringae* pv. tomato (*Pst*) DC3000 [21]. Similarly, *SlWRKY8* (*Solanum lycopersicum*, Sl) is positively involved in disease resistance against *Pst* DC3000 via activating the transcription levels of the *SlPR1a1* and *SlPR7* [22]. Interesting, *PtrWRKY18* (*Populus trichocarpa*, Ptr) and *PtrWRKY35* activate pathogenesis-related (PR) genes, and increase the resistance to the biotrophic pathogen *Melampsora* [23]. In the banana, *MaWRKY69* and *MaWRKY92* are highly upregulated in the susceptible cultivar, but downregulated in the resistant cultivar after infection by the root lesion nematode *Pratylenchus coffeae* [24]. *MaWRKY71*-overexpressing transgenic bananas have increased salt and oxidative stress resistance compared to wild-type bananas, with no effect on the disease resistance against *Foc* [25]. Additionally, *MaWRKY1/2* can directly bind to the promoters of *MaPR1-1*, *MaPR2*, and *MaPR10c* to regulate SA- and methyl jasmonate-induced pathogen resistance [10,26]. A recent study showed that *MaWRKY31*, *MaWRKY33*, *MaWRKY60*, and *MaWRKY71* directly bind to the W-box elements in the promoters of 9-cis-epoxycarotenoid dioxygenase 1 (*MaNCED1*) and *MaNCED2* and activate their expression, thereby regulating abscisic acid-induced cold tolerance in banana fruit [27]. Although *MaWRKYs* are comprehensively identified in the banana, the in vivo roles remain elusive [9,24], and need to be investigated further.

In this study, gene expression analysis revealed that some *MaWRKYs* might be involved in the defense response to fungal pathogen *Foc*. Further analysis revealed the subcellular location and transcription-activated activity on the W-box (TTGACC/T) of *MaWRKY24*. Notably, the transcriptional activation of *MaWRKY24* on several autophagy-associated genes (ATGs, *MaATG8f/g*) and their roles in plant disease susceptibility to soil-borne *Foc* were also highlighted. This study provided novel insight into *MaWRKY24i* mediated autophagy in soil-borne disease susceptibility.

## 2. Materials and Methods

### 2.1. Plant Materials and Growth Conditions

The banana variety BaXi jiao (BX) was used in this study. Tissue culture seedlings of bananas from the Tropical Seedling Tissue Culture Center (Danzhou, Hainan, China) were cultivated in Murashige and Skoog (MS) in the greenhouse, with 12 h light/ dark cycles and 120–150 µmol quanta m^-2^ s^-1^ irradiance at 26 °C.

The *Arabidopsis* seeds were sterilized by 70% ethanol for 1 min, followed by 4% NaClO for 5 min, and then washed at least five times with sterile, distilled water. After being kept at 4 °C in the dark for 24 h, they were placed on plates containing MS medium with 2% sucrose (pH 5.8). *Arabidopsis* seedlings were cultivated in a greenhouse (12 h light/ dark cycles and 120–150 µmol quanta m^-2^ s^-1^ irradiance at 22 °C).

*Foc* 4 was cultured on potato dextrose agar (PDA) medium for 7 d at 28 °C in the dark, and then fresh *Foc* 4 was washed out using sterilized water. The washed *Foc* 4 solution was filtered by sterilized six-layer gauze to remove the mycelium, and the spore solution concentration was adjusted to 5 × 10^6^ spores/mL for pathogen inoculation [6].

### 2.2. Phylogenetic Analysis of MaWRKYs

Obtaining sequences of 147 *MaWRKYs* and 82 *AtWRKYs* from Phytozome v12.1 (https://phytozome.jgi.doe.gov/pz/portal.html) and the *Arabidopsis* Information Resource (TAIR) version 10 (https://www.arabidopsis.org/), respectively, then the corresponding phylogenetic tree was structured by using Clustalx 1.83 and MEGA5.05 [28].

### 2.3. RNA Isolation and Quantitative Real-Time PCR (qRT-PCR)

RNA isolation was performed using a kit (DP441, TIANGEN, Beijing, China), and qRT-PCR was performed using the LightCycler^®^ 96 Real-Time PCR System (Roche, Basel, Switzerland), as Wei et al. [29] previously described. qRT-PCR profiles were determined following the protocol: 95 °C for 10 min, 45 cycles of 95 °C for 30 s, 55 °C for 30 s, and 72 °C for 20 s. Based on the Ct values, the corresponding gene expression levels were analyzed with the comparative 2^(-ΔΔCt)^ method [23]. The primers are listed in Table S1.

### 2.4. Subcellular Localization Analysis

The coding sequences of *MaWRKY24* were cloned into pEGAD vector [30] to form the constructions of *35S::GFP-MaWRKY24*. The primers are listed in Table S2. Subsequently, the GV3101 strains harboring an empty vector or the recombinant plasmids were syringe-infiltrated into *Nicotiana benthamiana* leaves, as previously described by Sparkes et al. [31]. Two days post-infiltration, the GFP signals of *35S::GFP* and *35S::GFP-MaWRKY24* and 4′, 6-diamidino-2-phenylindole (DAPI)-stained cell nuclei were detected via a confocal laser-scanning microscope (TCS SP8, Leica, Heidelberg, Germany).

### 2.5. Dural Luciferase (LUC) Assay through Transient Expression

5×TTGACC/T (W-box) and the promoter sequences of *MaATG8s* were cloned into the pGreenII 0800-LUC vector to form the constructions of *35S::REN-*5×W-box/*proMaATG8s-LUC*. The primers are listed in Table S2. After 12 h of transformation in leaf protoplasts, as Yoo et al. [32] previously described, the LUC and REN were quantified in the transformed protoplasts using a dual luciferase reporter gene assay kit (RG027, Beyotime, Haimen, Jiangsu, China).

### 2.6. Chromatin Immunoprecipitation Quantitative Real-Time PCR (ChIP-qPCR)

Banana protoplast isolation was performed according to Sagi et al. [33]. The banana nucleus was extracted using Plant Nuclei Isolation/Extraction Kit (CELLYTPN1, Sigma, Missouri, USA) according to the instruction. Then each sample was divided into two parts, protein A/G (C40091707, GenScript) and GFP antibody (G1546, Sigma) or IgG (A4416, Sigma) was added, and the solution was mixed and incubated for 6 h at 4 °C. DNA isolation was performed using a kit (32817KC1, AXYGEN), and qRT-PCR was performed. The primers are listed in Table S1.

### 2.7. Electrophoretic Mobility Shift Assay (EMSA)

The coding sequences of *MaWRKY24* was cloned into the pET28a vector to form the constructions of *pET28a-MaWRKY24*. The primers are listed in Table S1. The BL21 strain harboring *pET28a-MaWRKY24* was induced by 1 mM IPTG for protein expression and purification (His-tag Protein Purification Kit, P2226, Beyotime, Haimen, Jiangsu, China), and the synthesized double-stranded probes were used for EMSA, as Ream et al. [34] previously described.

### 2.8. Generation of Transgenic Plants and Observation of the Autophagosome

The coding sequences of *MaATG8f/g* have been cloned into the pEGAD vector [30] as per a previous work [29]. Then the recombinant plasmids (*35S::GFP-MaWRKY24*, *35S::GFP*-*MaATG8f* and *35S::GFP*-*MaATG8g*) were transformed into *Agrobacterium tumefaciens* GV3101, which was further used to obtain transgenic *Arabidopsis* plants via the floral dip method [35,36]. The positive transgenic plants were chosen by Basta resistance and confirmed by PCR. For the observation of autophagosomes, 14-day-old seedlings of wild-type (WT) and *MaATG8f/g*-overexpressing lines were transferred into a MS medium with or without *Foc* 4 spore solution for 8 h. Thereafter, the GFP fluorescence in *Arabidopsis* roots was detected using biotechnical microscopy (DM5000, Leica, Wetzlar, Germany).

### 2.9. Statistical Analysis 

In this study, at least three biological replicates were carried out for all experiments, and the average means ± SD are shown. After comprehensive analysis by Duncan’s range test using Statistical Analysis System (SAS) v9.4 software (SAS Instituteinc, North Carolina, USA), asterisks (*) show significant difference at *p* < 0.05.

## 3. Results

### 3.1. Evolutionary Analysis and Expression Profiles of MaWRKYs

**Figure 1:**
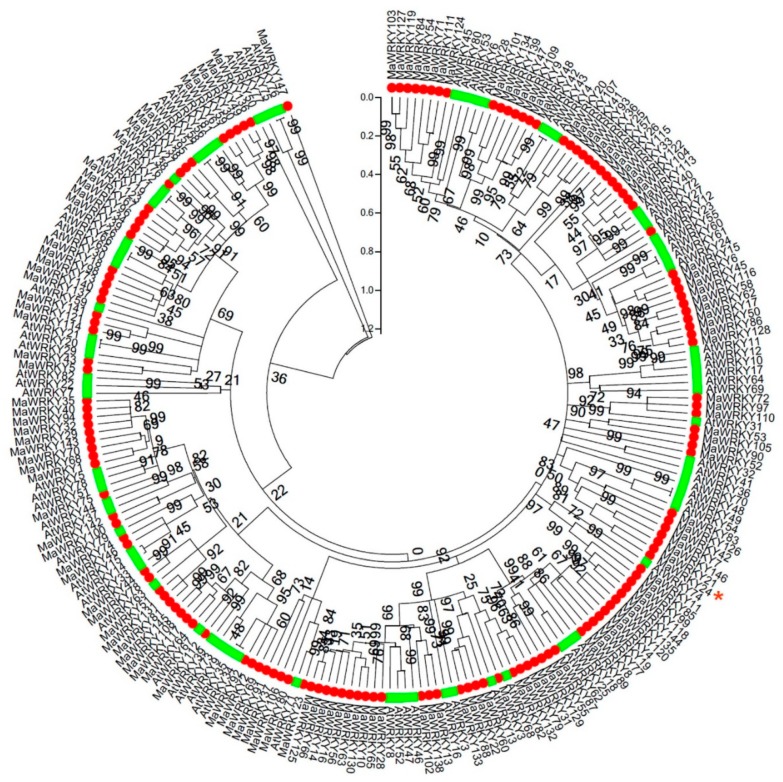
A neighbor-joining phylogenetic tree was constructed to investigate the evolution of *MaWRKYs* and *AtWRKYs*. The green and red shapes show *AtWRKYs* and *MaWRKYs*, respectively. The phylogenetic tree was established on the basis of the coding sequence using Clustalx 1.83 and MEGA5.05. The red asterisk indicates the location of *MaWRKY24*.

**Figure 2:**
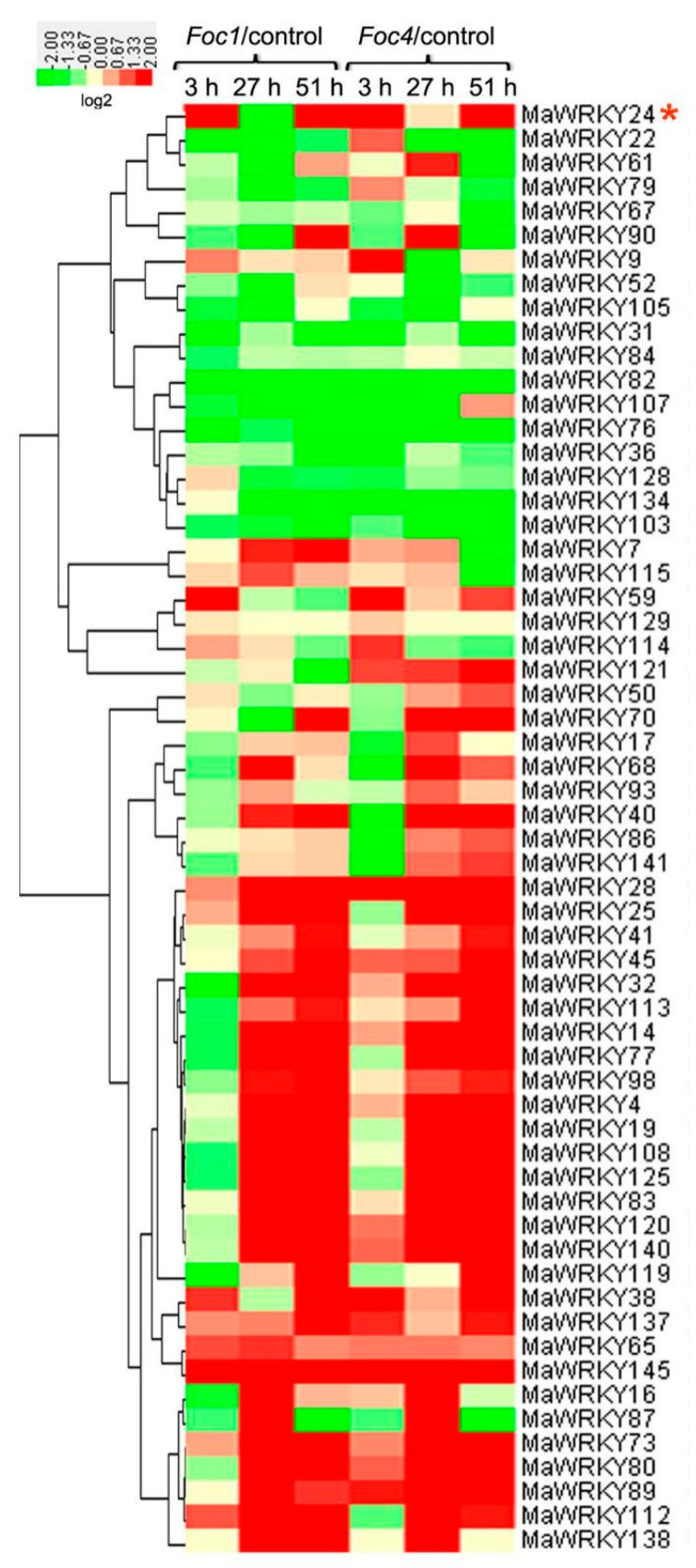
The significant relative transcription levels of *MaWRKYs* in response to *Foc* 1 and *Foc* 4. The values of transcriptomic data of corresponding genes were downloaded from the Supplementary Table S4 in Li et al. [2], and have also described in the Supplementary Table 5 in Goel et al. [9]. In the assay, banana roots were inoculated by the control or *Foc 1* or *Foc* 4 for 3 h, 27 h, or 51 h. A heatmap of gene expression profile was constructed using CLUSTER and Java Treeview. The red asterisk indicates the location of *MaWRKY24*.

To analyze the evolutionary relationships between MaWRKYs and AtWKRYs, an unrooted neighbor-joining phylogenetic tree was constructed based on the predicted amino acid sequences. As shown in Figure 1, a neighbor-joining phylogenetic tree, with five groups, was constructed to investigate the evolution between 147 *MaWRKYs* and 82 *AtWRKYs*. The groups contained 39, 14, 42, 27, and 25 MaWRKYs. Among them, MaWRKY24 belonged to the second group, and showed high homology with AtWRKY48, AtWRKY49, and AtWRKY54. Moreover, both AtWRKY48 and AtWRKY54 are negative regulators in plant basal defense response [37,38]. Based on previously published data [2], we analyzed the transcript profiles of *MaWRKYs* in banana roots’ response to *Foc* 1/4 infection. In brief, 60 of 147 *MaWRKYs* were significantly induced in response to *Foc* infection, with at least 2-fold changes (Figure 2). Some *MaWRKYs*, such as *MaWRKY28/73/145*, were generally upregulated by *Foc* 1/4 infection, while some *MaWRKYs* (*MaWRKY76*, *82*, *84*, *103*, and so on) were commonly downregulated by *Foc* 1/4 infection (Figure 2). Interestingly, *MaWRKY24* was significantly upregulated at 3 and 51 h, but downregulated at 27 h by *Foc* 1/4. According to Li et al. [2], 27 h post-inoculation, spores and hyphae are attached to the banana roots inoculated with *Foc* ¼; 51 h post-inoculation, hyphae spread into the vascular tissues of the roots infected with *Foc* 1/4. Therefore, the induction of *MaWRKY24* might be involved in early and later stages of banana‒*Foc* 1/4 interaction, and the dual transcriptional changes of *MaWRKY24* also indicate its precise modulation in the banana‒*Foc* 1/4 interaction. In addition, MaWRKY24 showed high homology with AtWRKY48 and AtWRKY54, which are negative regulators of plant basal defense response [37,38]. Therefore, *MaWRKY24* was selected for further functional analysis.

### 3.2. Subcellular Localization and Transcriptional Activated Activity of MaWRKY24

**Figure 3:**
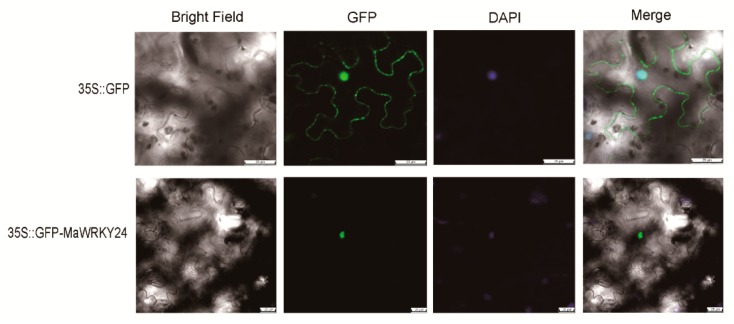
Subcellular localization of MaWRKY24. The GFP signals of *35S::GFP* and *35S::GFP-MaWRKY24* and DAPI-stained cell nuclei are shown. Bar = 25 μm.

**Figure 4:**
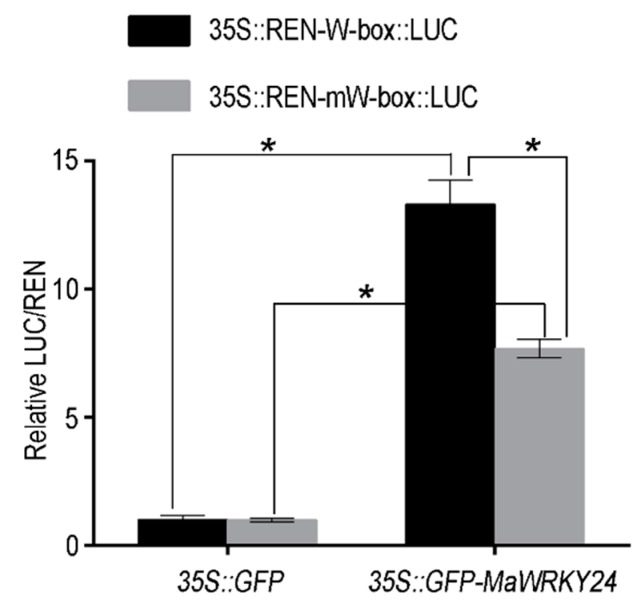
The transcriptional activated activity of *MaWRKY24*. In the dual LUC assay in plant leaf protoplasts, *35S::GFP* and *35S::GFP-MaWRKY24* were used as the effectors, and *35S::REN-*5×W-box*-LUC* was used as the reporter. Asterisks (*) show significant difference at *p* < 0.05.

In the transient *N*. *benthamiana* leaves, the fluorescence of *35S::GFP* was located in both the cytoplasm and nucleus, while that of *35S::GFP-MaWRKY24* was specifically colocalized with DAPI-stained nucleus (Figure 3). As transcription factors, plant WRKYs are widely recognized as binding to the W-box (5’-TTGACC/T-3’). Using dual LUC assay in plant leaf protoplasts, the overexpression of *MaWRKY24* significantly activated the LUC of 5×W-box-pGreenII 0800-LUC (Figure 4). Therefore, the specific nucleus subcellular location and transcription-activated activity on the W-box indicated that *MaWRKY24* was a transcription factor.

### 3.3. Overexpression of MaWRKY24 Regulated Expression Level of MaATG8s

**Figure 5:**
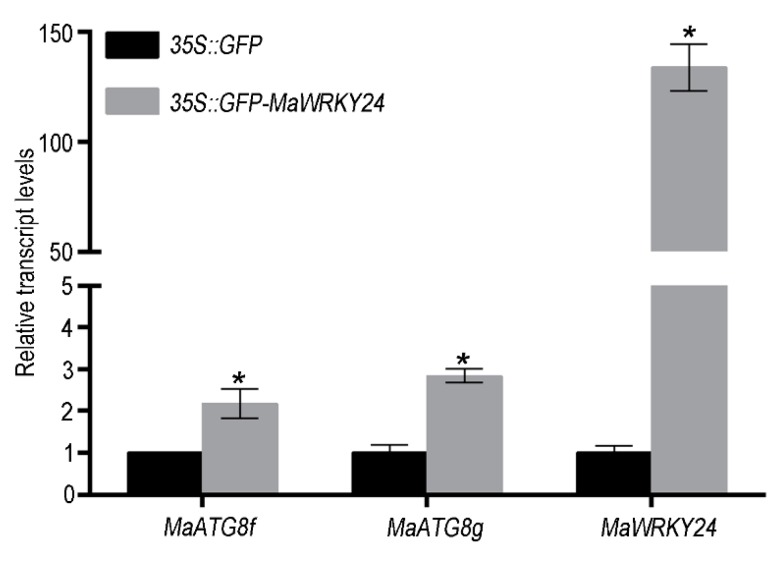
The effect of *MaWRKY24* overexpression on the relative transcription levels of *MaATG8f/g* in leaf protoplasts. Asterisks (*) show significant difference at *p* < 0.05.

In previous studies, we found that *MaATG8s* were also commonly regulated by *Foc* 1 and *Foc* 4 [29]. Interestingly, the promoters of some *MaATG8s* are widely distributed with the W-box. To investigate the relationship between these *MaWRKYs* and *MaATG8s*, we performed a cluster analysis of the correlation between *MaWRKYs* and *MaATG8s* expression. The result showed that the expression of *MaWRKY24* was most closely related to that of *MaATG8f/g* (Figure S1). Consistently, we found that the relative transcriptional levels of *MaATG8f/g* were significantly upregulated in *MaWRKY24*-overexpressing protoplasts (Figure 5), indicating that *MaWRKY24* could activate the expression of *MaATG8f/g*.

### 3.4. MaWRKY24 were Transcriptional Activators of MaATG8f/g

**Figure 6:**
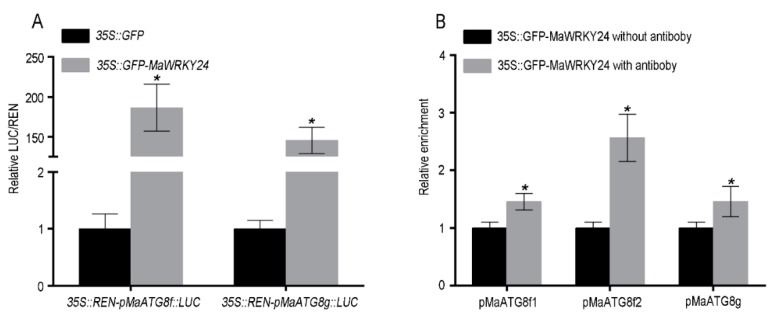
*MaWRKY24* as a transcriptional activator of *MaATG8f/g*. (**A**) In the dual LUC assay, *35S::GFP* and *35S::GFP-MaWRKY24* were used as the effectors, and 35S::REN-*pMaATG8s*-LUC was used as the reporter in plant leaf protoplasts. (**B**) Analysis of the relative enrichment of the *MaATG8s* promoter by ChIP-qPCR in banana leaf protoplasts. Asterisks (*) show significant difference at *p <* 0.05.

In addition, we investigated the direct effect of *MaWRKY24* overexpression on the promoter activities of *MaATG8f/g*. Dual LUC assay in leaf protoplasts showed that overexpression of *MaWRKY24* significantly activated the LUC of *MaATG8f/g* promoters (Figure 6A). To determine whether *MaWRKY24* combined with the W-box element, ChIP-qPCR analysis was performed. The result showed that the relative enrichment of promoter regions of *MaATG8f/g* with W-box was higher than that of control (Figure 6B).

**Figure 7:**
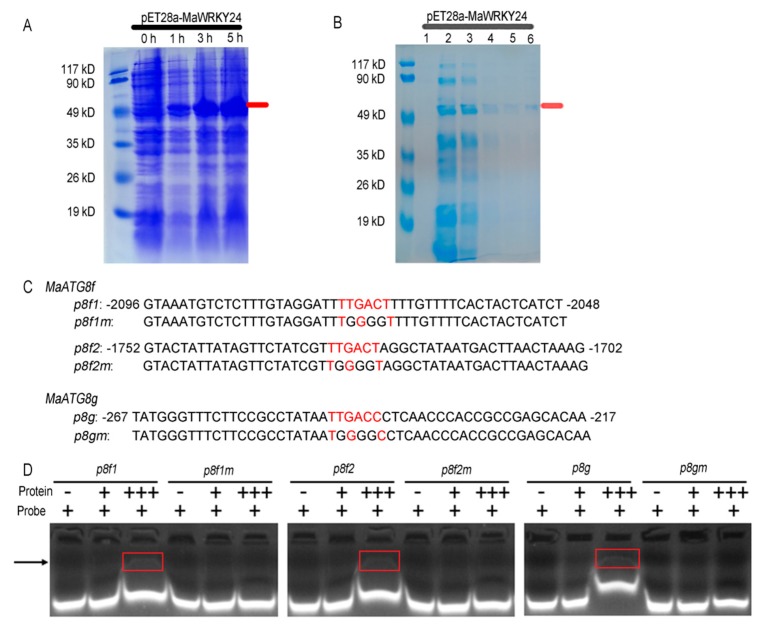
Assay of the interaction between MaWRKY24 and MaATG8f/g promoters. (**A**) MaWRKY24-pET28a induced expression by SDS-PAGE. The red line indicates the induced recombinant protein. (**B**) MaWRKY24-pET28a was purified. 1‒6 indicate the first to sixth tubes of the collected purified protein by elution. The red line indicates the corresponding induced recombinant protein. (**C**) The probe sequences of the wild type and the mutation. (**D**) The binding of *MaWRKY24* to the W-box of *MaATG8f/g* promoters through EMSA. The arrow and square frame indicate the probe‒protein complex.

To confirm the direct binding of *MaWRKY24* to the promoter region of *MaATG8f/g* with the W-box, the protein of MaWRKY24 was induced and purified for EMSA analysis. The result confirmed that *MaWRKY24* direct binding to the corresponding probes containing W-box in *MaATG8f/g* promoters in vitro (Figure 7). In summary, these results suggested that *MaWRKY24* bound to the W-box regions in the promoter of *MaATG8f/g* and served as a transcriptional activator of *MaATG8f/g*.

### 3.5. Overexpression of MaWRKY24 and MaATG8f/g Negatively Regulate Plant Disease Susceptibility to Foc 4

**Figure 8:**
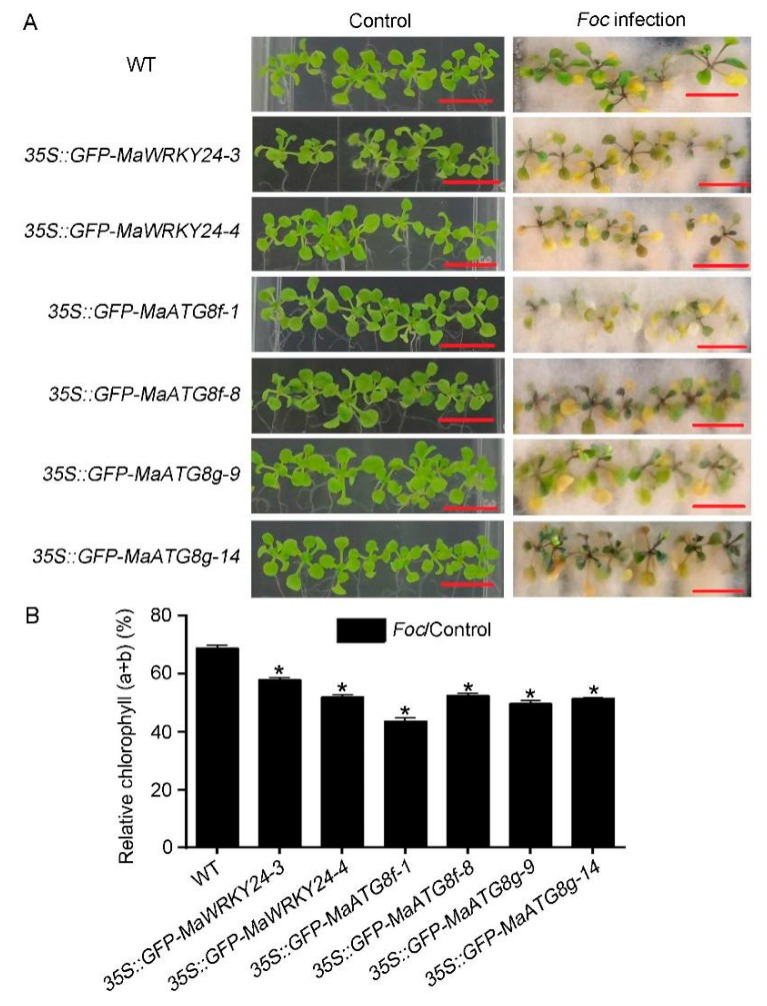
Phenotypes of WT and transgenic seedlings in response to *Foc* 4 infection. (**A**) The phenotypes of seedlings under control and *Foc* 4 infection conditions. Fourteen-day-old seedlings of WT, *MaWRKY24*- and *MaATG8f/g*-overexpressing lines were without or infected with *Foc* 4 spore solution for 7 d and then the phenotype was observed. Bar = 1 cm. (**B**) Relative chlorophyll (a + b) of WT and transgenic seedlings. Asterisks (*) show significant difference at *p <* 0.05.

To further study the roles of *MaWRKY24* and *MaATG8f/g* in response to *Foc* 4 infection, 14-day-old *MaWRKY24* and *MaATG8f/g* overexpressing *Arabidopsis* seedlings were grown under control and *Foc* 4 infection conditions. Under control conditions, there were no significant differences in phenotype between the WT and transgenic lines (Figure 8A–B). Under *Foc* 4 infection, both *MaWRKY24*- and *MaATG8f/g*-overexpressing *Arabidopsis* seedlings showed worse growth and lower chlorophyll (a+b) content than WT (Figure 8A–B). Therefore, overexpression of *MaWRKY24* and *MaATG8f/g* might negatively regulate plant disease susceptibility to *Foc* 4 infection in *Arabidopsis*.

**Figure 9:**
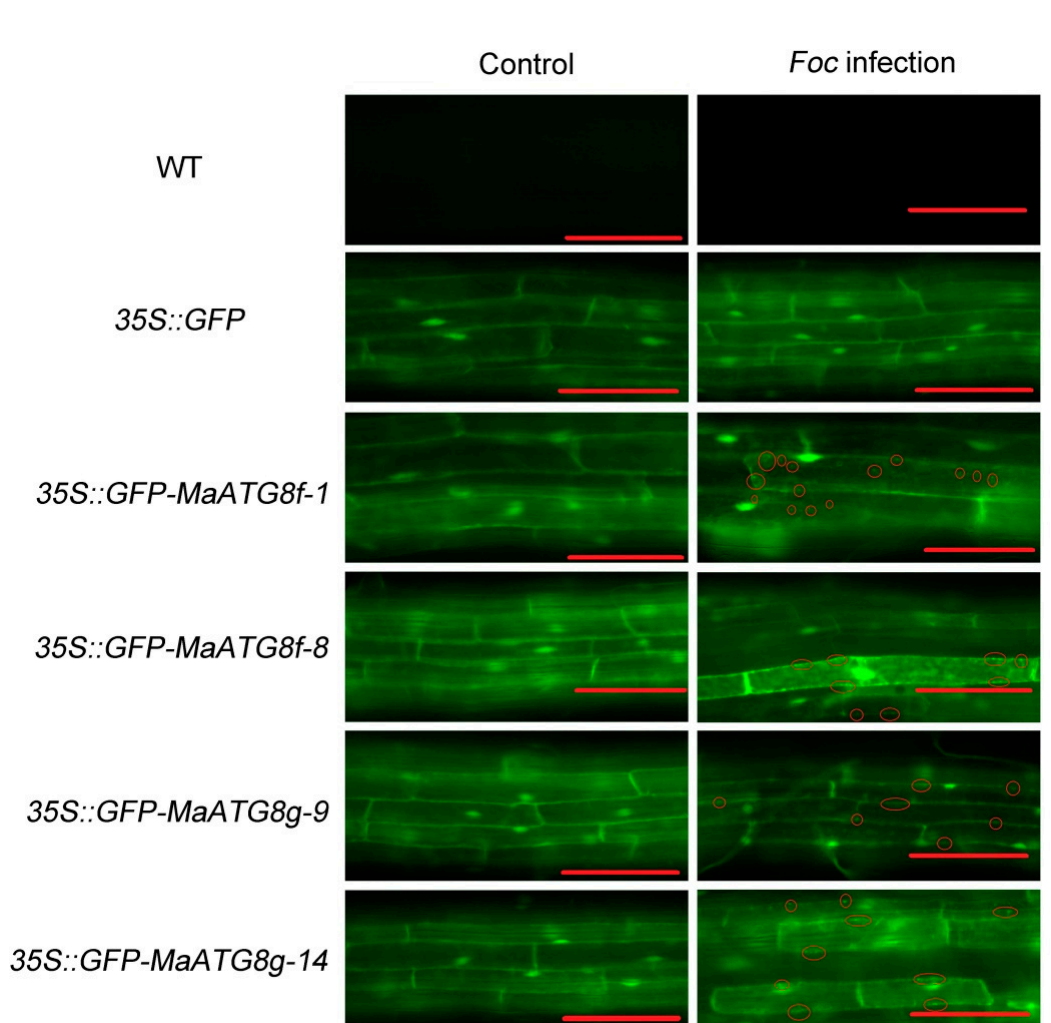
The effect of *Foc* 4 on the formation of autophagosomes in *Arabidopsis* seedling roots. For the examination, 14-day-old seedlings of WT, *35S::GFP* and *MaATG8f/g* overexpressing lines were transferred into MS medium with or without *Foc* 4 spores solution for 8 h and then the autophagosomes were examined. Autophagosomes were marked with circles. Bar = 100 μm.

To investigate whether *Foc* 4 affected autophagy in *Arabidopsis* roots, the roots of WT, *35S::GFP* and *35S:GFP-MaATG8f/g* seedlings were infected with a *Foc* 4 spore solution for 8 h. The green fluorescent spots indicated that autophagosomes were stronger after *Foc* 4 infection in *MaATG8f/g* transgenic *Arabidopsis*, but not in the WT and the *35S::GFP* transgenic line (Figure 9). The results indicated that *Foc* 4 might induce the formation of autophagosomes.

## 4. Discussion and Conclusions

Although the banana is one of the most popular fresh fruits in the world, it is seriously affected by *Foc* in subtropical and tropical areas [2]. Furthermore, no strong and continuous disease-resistant banana variety is effective in the banana cultivation [39]. Therefore, it is essential to construct strong disease-resistant banana varieties through molecular and genetic breeding.

WRKY transcription factors are widely involved in plant stress responses [18,40,41,42,43,44]. In plants, most WRKY transcription factors are activated by plant‒pathogen interaction, and regulate multiple downstream genes via binding to W-box elements in the promoter [45,46,47]. Herein, we found that 60 of 147 *MaWRKYs* were significantly affected by *Foc* infection. Additionally, the overexpression of *MaWRKY24* negatively regulates plant disease susceptibility to *Foc* 4. In *Arabidopsis*, flg22 induces the transcripts of *AtWRKY18*, *AtWRKY33*, and *AtWRKY40*, resulting in the activation of hundreds of genes with W-box elements [45]. *AtWRKY8* has a positive effect on the resistance to *Pseudomonas syringae, B. cinerea*, and salinity stress [48,49]. Consistently, *SlWRKY8* functions as a positive regulator in resisting *Pst* DC3000 [22]. However, *AtWRKY18* and *AtWRKY40* act as negative resistance against the obligate hemibiotrophic fungus *Golovinomyces orontii* [50], and *PtrWRKY40* also negatively regulates disease resistance against *Dothiorella gregaria* in the poplar [51]. Similarly, overexpression of *GhWRKY25* enhances disease susceptibility to *Botrytis cinerea* by inhibiting the expression of SA or ethylene signaling-related genes [52]. It is widely known that the conservation of the WRKY domain can recognize and bind with the W-box [10,53]. We also found that the overexpression of *MaWRKY24* could significantly recognize and bind with the W-box. For example, *PcWRKY1*, a transcriptional activator, mediates fungal elicitor-induced gene expression by binding to W-box elements [54]. *HvWRKY38* binding to W-box elements are involved in cold and drought response [55]. In this study, *MaWRKY24* had significant effects on the W-box, together with the specific nucleus subcellular location, indicating that it is a real WRKY transcript factor.

As the key regulator of cellular homeostasis, autophagy is a transport pathway that mediates the transfer and degradation of cytoplasmic materials [56]. Briefly, autophagy breaks down the damaged cytoplasmic constituents in a cell and recycles the cellular cytoplasmic components [57]. Autophagy is coordinated by evolutionarily conserved ATGs that are essential for biotic and abiotic stress responses [58,59,60,61,62]. For example, autophagic activity is induced by necrotrophic fungal pathogens [63,64]. Herein, the green fluorescence spots in *MaATG8f/g*-overexpressing lines were stronger after *Foc* 4 spore solution treatment compared to the control, indicating the activation of autophagosomes and autophagic activity by *Foc* 4. Meanwhile, *MaATG8f/g* played a negative role in disease susceptibility to *Foc* 4. Recently, we have highlighted the effects of *MaATG8s* on hypersensitive-like cell death and immune responses, which are directly related to autophagy [29]. Moreover, ATG8s are central parts of the latter process among autophagy-related proteins [58]. The results showed that the expression of *MaWRKY24* was closely related to that of *MaATG8f/g*. *MaWRKY24* was the transcriptional activator of *MaATG8f/g*, due to the direct binding of *MaWRKY24* to the W-box in the promoter of *MaATG8f/g*. *AtWRKY33* interacts with *ATG18a* and both are involved in plant responses to necrotizing trophic pathogens [65]. Previous studies have shown that the crosstalk of WRKYs and ATGs as well as autophagy may be involved in plant resistance to necrotrophic pathogens and bacterial pathogens [65,66,67].

**Figure 10:**
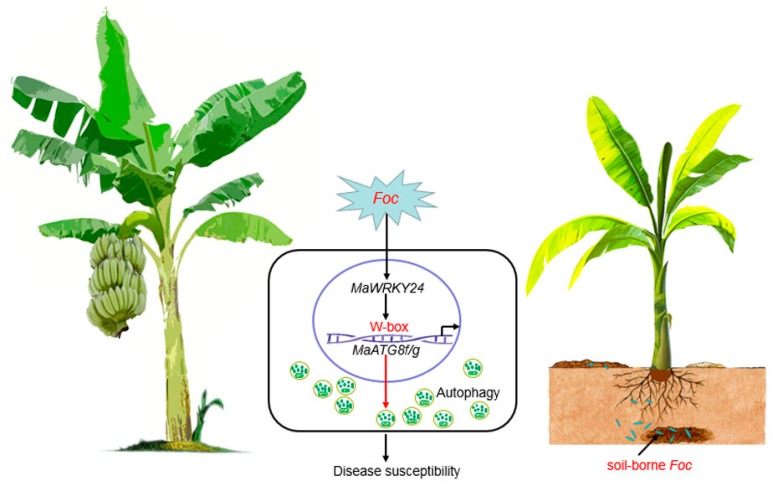
A model for the correlation between *MaWRKY24* and autophagy in the banana.

This study highlights the positive regulation of *MaWRKY24* in the transcriptional activation of *MaATG8f/g* and their common roles in plant disease susceptibility to *Foc* 4, indicating the correlation between *MaWRKY24*, autophagy, and disease susceptibility (Figure 10).

## Supplementary Materials

The following are available online https://www.mdpi.com/2076-0817/8/4/264/s1. Table S1: The primers were used for the quantitative real time PCR. Table S2: The primers were used for the vector construction. Figure S1: Cluster analysis of correlation between *MaWRKYs* and *MaATG8s* expression.

## References

[1] Di X., Takken F., Tintor N. (2016). How phytohormones shape interactions between plants and the soil-borne fungus *Fusarium oxysporum*. Front. Plant Sci..

[2] Li C., Shao J., Wang Y., Li W., Guo D., Yan B., Xia Y., Peng M. (2013). Analysis of banana transcriptome and global gene expression profiles in banana roots in response to infection by race 1 and tropical race 4 of *Fusarium oxysporum* f. sp. *cubense*. BMC Genom..

[3] Berg N., Berger D., Hein I., Birch P., Wingfield M., Viljoen A. (2007). Tolerance in banana to *Fusarium* wilt is associated with early up-regulation of cell wall-strengthening genes in the roots. Mol. Plant Pathol..

[4] Ordonez N., Seidl M., Waalwijk C., Drenth A., Kilian A., Thomma B., Ploetz R., Kema G. (2015). Worse comes to worst: Bananas and panama disease-when plant and pathogen clones meet. PLoS Pathog..

[5] Ploetz R. (2015). Fusarium wilt of banana. Phytopathology.

[6] Cheng C., Liu F., Sun X., Tian N., Mensah R., Li D., Lai Z. (2019). Identification of *Fusarium oxysporum* f. sp. *cubense* tropical race 4 (*Foc* TR4) responsive miRNAs in banana root. Sci. Rep..

[7] Thangavelu R., Palaniswami A., Velazhahan R. (2004). Mass production of *Trichoderma harzianum* for managing Fusarium wilt of banana. Agric. Ecosyst. Environ..

[8] Dong X., Ling N., Wang M., Shen Q., Guo S. (2012). Fusaric acid is a crucial factor in the disturbance of leaf water imbalance in *Fusarium*-infected banana plants. Plant Physiol. Biochem..

[9] Goel R., Pandey A., Trivedi P., Asif M. (2016). Genome-wide analysis of the Musa WRKY gene family: Evolution and differential expression during development and stress. Front. Plant Sci..

[10] Rushton P., Somssich I., Ringler P., Shen Q. (2010). WRKY transcription factors. Trends Plant Sci..

[11] Wei Y., Shi H., Xia Z., Tie W., Ding Z., Yan Y., Wang W., Hu W., Li K. (2016). Genome-Wide identification and expression analysis of the WRKY gene family in cassava. Front. Plant Sci..

[12] Luo Y., Yu S., Li J., Li Q., Wang K., Huang J., Liu Z. (2018). Molecular characterization of WRKY transcription factors that act as negative regulators of O-methylated catechins biosynthesis in tea plant (*Camellia Sinensis* L.). J. Agric. Food Chem..

[13] Akagi A., Fukushima S., Okada K., Jiang C., Yoshida R., Nakayama A., Shimono M., Sugano S., Yamane H., Takatsuji H. (2014). WRKY45-Dependent priming of diterpenoid phytoalexin biosynthesis in rice and the role of cytokinin in triggering the reaction. Plant Mol. Biol..

[14] Mao G., Meng X., Liu Y., Zheng Z., Chen Z., Zhang S. (2011). Phosphorylation of a WRKY transcription factor by two pathogen-responsive MAPKs drives phytoalexin biosynthesis in *Arabidopsis*. Plant Cell.

[15] Zheng Y., Deng X., Qu A., Zhang M., Tao Y., Yang L., Liu Y., Xu J., Zhang S. (2018). Regulation of pollen lipid body biogenesis by MAP kinases and downstream WRKY transcription factors in *Arabidopsis*. PLoS Genet..

[16] Xu J., Zhang S. (2015). Mitogen-Activated protein kinase cascades in signaling plant growth and development. Trends Plant Sci..

[17] Guan Y., Meng X., Khanna R., LaMontagne E., Liu Y., Zhang S. (2014). Phosphorylation of a WRKY transcription factor by MAPKs is required for pollen development and function in *Arabidopsis*. PLoS Genet..

[18] Adachi H., Nakano T., Miyagawa N., Ishihama N., Yoshioka M., Katou Y., Yaeno T., Shirau K., Yoshioka H. (2015). WRKY transcription factors phosphorylated by MAPK regulate a plant immune NADPH oxidase in *Nicotiana benthamiana*. Plant Cell.

[19] Chen L., Xiang S., Chen Y., Li D., Yu D. (2017). *Arabidopsis* WRKY45 interacts with the DELLA protein RGL1 to positively regulate age-triggered leaf senescence. Mol. Plant.

[20] Miao Y., Zentgraf U. (2007). The antagonist function of *Arabidopsis* WRKY53 and ESR/ESP in leaf senescence is modulated by the jasmonic and salicylic acid equilibrium. Plant Cell.

[21] Wang X., Guo R., Tu M., Wang D., Guo C., Wan R., Li Z., Wang X. (2017). Ectopic expression of the wild grape WRKY transcription factor VqWRKY52 in *Arabidopsis thaliana* enhances resistance to the biotrophic pathogen powdery mildew but not to the Necrotrophic Pathogen *Botrytis cinerea*. Front. Plant Sci..

[22] Gao Y., Liu J., Yang F., Zhang G., Wang D., Zhang L., Ou Y., Yao Y. (2019). The WRKY transcription factor WRKY8 promotes resistance to pathogen infection and mediates drought and salt stress tolerance in *Solanum lycopersicum*. Physiol. Plant.

[23] Jiang Y., Guo L., Ma X., Zhao X., Jiao B., Li C., Luo K. (2017). The WRKY transcription factors PtrWRKY18 and PtrWRKY35 promote *Melampsora* resistance in *Populus*. Tree Physiol..

[24] Kaliyappan R., Viswanathan S., Suthanthiram B., Subbaraya U., Marimuthu S., Muthu M. (2016). Evolutionary expansion of WRKY gene family in banana and its expression profile during the infection of root lesion nematode, *Pratylenchus coffeae*. PLoS ONE.

[25] Shekhawat U., Ganapathi T. (2013). *MusaWRKY71* overexpression in banana plants leads to altered abiotic and biotic stress responses. PLoS ONE.

[26] Tang Y., Kuang J., Wang F., Chen L., Hong K., Xiao Y., Xie H., Lu W., Chen J. (2013). Molecular characterization of *PR* and WRKY genes during SA- and MeJA-induced resistance against *Colletotrichum musae* in banana fruit. Postharvest Biol. Technol..

[27] Luo D., Ba L., Shan W., Kuang J., Lu W., Chen J. (2017). Involvement of WRKY transcription factors in abscisic-acid-induced cold tolerance of banana fruit. J. Agric. Food Chem..

[28] Tamura K., Peterson D., Peterson N., Stecher G., Nei M., Kumar S. (2011). MEGA5: Molecular evolutionary genetics analysis using maximum likelihood, evolutionary distance, and maximum parsimony methods. Mol. Biol. Evol..

[29] Wei Y., Liu W., Hu W., Liu G., Wu C., Liu W., Zeng H., He C., Shi H. (2017). Genome-Wide analysis of autophagy-related genes (ATGs) in banana highlights *MaATG8s* in cell death and autophagy in immune response to Fusarium wilt. Plant Cell Rep..

[30] Cutler S., Ehrhardt D., Griffitts J., Somerville C. (2000). Random GFP: cDNA fusions enable visualization of subcellular structures in cells of *Arabidopsis* at a high frequency. Proc. Natl. Acad. Sci. USA.

[31] Sparkes I., Runions J., Kearns A., Hawes C. (2006). Rapid, transient expression of fluorescent fusion proteins in tobacco plants and generation of stably transformed plants. Nat. Protoc..

[32] Yoo S., Cho Y., Sheen J. (2007). *Arabidopsis* mesophyll protoplasts: A versatile cell system for transient gene expression analysis. Nat. Protoc..

[33] Sagi L., Remy S., Panis B., Swennen R., Volckaert G. (1994). Transient gene expression in electroporated banana (*Musa* spp., cv. ‘Bluggoe’, ABB group) protoplasts isolated from regenerable embryogenetic cell suspensions. Plant Cell Rep..

[34] Ream J., Lewis L., Lewis K. (2016). Rapid agarose gel electrophoretic mobility shift assay for quantitating protein: RNA interactions. Anal. Biochem..

[35] Clough S., Bent A. (1998). Floral dip: A simplified method for agrobacterium-mediated transformation of *Arabidopsis thaliana*. Plant J..

[36] Li B., Liu G. (2020). Construction of overexpressing *MaATG8g Arabidopsis* and analysis of the promoter. https://kns.cnki.net/KCMS/detail/46.1068.S.20191008.1121.008.html.

[37] Xing D., Lai Z., Zheng Z., Vinod K., Fan B., Chen Z. (2008). Stress- and pathogen-induced *Arabidopsis* WRKY48 is a transcriptional activator that represses plant basal defense. Mol. Plant.

[38] Li J., Zhong R., Palva E. (2017). WRKY70 and its homolog WRKY54 negatively modulate the cell wall-associated defenses to necrotrophic pathogens in Arabidopsis. PLoS ONE.

[39] García-Bastidas F., Van der Veen A., Nakasato-Tagami G., Meijer H., Arango-Isaza R., Kema G. (2019). An improved phenotyping protocol for panama disease in banana. Front. Plant Sci..

[40] Cheng H., Liu H., Deng Y., Xiao J., Li X., Wang S. (2015). The WRKY45-2 WRKY13 WRKY42 transcriptional regulatory cascade is required for rice resistance to fungal pathogen. Plant Physiol..

[41] Jiang Y., Yu D. (2015). WRKY transcription factors: Links between phytohormones and plant processes. Sci. China Life Sci..

[42] Chen L., Zhang L., Li D., Wang F., Yu D. (2013). WRKY8 transcription factor functions in the TMV-cg defense response by mediating both abscisic acid and ethylene signaling in *Arabidopsis*. Proc. Natl. Acad. Sci. USA.

[43] Kim K., Lai Z., Fan B., Chen Z. (2008). *Arabidopsis* WRKY38 and WRKY62 transcription factors interact with histone deacetylase 19 in basal defense. Plant Cell.

[44] Lai Z., Li Y., Wang F., Cheng Y., Fan B., Yu J., Chen Z. (2011). Arabidopsis sigma factor binding proteins are activators of the WRKY33 transcription factor in plant defense. Plant Cell.

[45] Birkenbihl R., Kracher B., Somssich I. (2017). Induced genome-wide binding of three Arabidopsis WRKY transcription factors during early MAMP-triggered immunity. Plant Cell.

[46] Buscaill P., Rivas S. (2014). Transcriptional control of plant defence responses. Curr. Opin. Plant Biol..

[47] Pandey S., Somssich I. (2009). The role of WRKY transcription factors in plant immunity. Plant Physiol..

[48] Chen L., Zhang L., Yu D. (2010). Wounding-Induced WRKY8 is involved in basal defense in *Arabidopsis*. Mol. Plant Microbe. Interact..

[49] Hu Y., Chen L., Wang H., Zhang L., Wang F., Yu D. (2013). Arabidopsis transcription factor WRKY8 functions antagonistically with its interacting partner VQ9 to modulate salinity stress tolerance. Plant J..

[50] Pandey S., Roccaro M., Schön M., Logemann E., Somssich I. (2010). Transcriptional reprogramming regulated by WRKY18 and WRKY40 facilitates powdery mildew infection of Arabidopsis. Plant J..

[51] Karim A., Jiang Y., Guo L., Ling Z., Ye S., Duan Y., Li C., Luo K. (2015). Isolation and characterization of a subgroup iia WRKY transcription factor PtrWRKY40 from *Populus trichocarpa*. Tree Physiol..

[52] Liu X., Song Y., Xing F., Wang N., Wen F., Zhu C. (2016). *GhWRKY25*, a group I *WRKY* gene from cotton, confers differential tolerance to abiotic and biotic stresses in transgenic *Nicotiana Benthamiana*. Protoplasma.

[53] Eulgem T., Rushton P., Robatzek S., Somssich I. (2000). The WRKY superfamily of plant transcription factors. Trends Plant Sci..

[54] Eulgem T. (1999). Early nuclear events in plant defence signalling: Rapid gene activation by WRKY transcription factors. EMBO J..

[55] Marè C., Mazzucotelli E., Crosatti C., Francia E., Stanca A., Cattivelli L. (2004). *Hv-WRKY38*: A new transcription factor involved in cold- and drought-response in barley. Plant Mol. Biol..

[56] Marshall R., Vierstra R. (2018). Autophagy: The master of bulk and selective recycling. Annu. Rev. Plant Biol..

[57] Thompson A., Vierstra R. (2005). Autophagic recycling: Lessons from yeast help define the process in plants. Curr. Opin. Plant Biol..

[58] Klionsky D., Abdalla F., Abeliovich H., Abraham R., Acevedo-Arozena A., Adeli K., Agholme L., Agnello M., Agostinis P., Aguirre-Ghiso J. (2012). Guidelines for the use and interpretation of assays for monitoring autophagy. Autophagy.

[59] Dagdas Y., Beihaj K., Maqbool A., Chaparro-Garcia A., Pandey P., Petre B., Tabassum N., Cruz-Mireles N., Hughes R., Sklenar J. (2016). An effector of the irish potato famine pathogen antagonizes a host autophagy cargo receptor. eLife.

[60] Avin-Wittenberg T., Baluška F., Bozhkov P., Elander P., Fernie A., Galili G., Hassan A., Hofius D., Isono E., Bars R. (2018). Autophagy-Related approaches for improving nutrient use efficiency and crop yield protection. J. Exp. Bot..

[61] Minina E., Moschou P., Bozhkov P. (2017). Limited and digestive proteolysis: Crosstalk between evolutionary conserved pathways. New Phytol..

[62] Ustun S., Hafren A., Liu Q., Marshall R., Minina E., Bozhkov P., Vierstra R., Hofius D. (2018). Bacteria exploit autophagy for proteasome degradation and enhanced virulence in plants. Plant Cell.

[63] Lenz H., Vierstra R., Nürnberger T., Gust A. (2011). *ATG7* contributes to plant basal immunity towards fungal infection. Plant Signal. Behav..

[64] Liu Y., Xiong Y., Bassham D. (2009). Autophagy is required for tolerance of drought and salt stress in plants. Autophagy.

[65] Lai Z., Wang F., Zheng Z., Fan B., Chen Z. (2011). A critical role of autophagy in plant resistance to necrotrophic fungal pathogens. Plant J..

[66] Yan Y., Wang P., He C., Shi H. (2017). *MeWRKY20* and its interacting and activating autophagy-related protein 8 (*MeATG8*) regulate plant disease resistance in cassava. Biochem. Biophys. Res. Commun..

[67] Zhou J., Yu J., Chen Z. (2014). The perplexing role of autophagy in plant innate immune responses. Mol. Plant Pathol..

