# Supplementary material for: Functional Analysis of MaWRKY24 in Transcriptional Activation of Autophagy-Related Gene 8f/g and Plant Disease Susceptibility to Soil-Borne Fusarium oxysporum f. sp. cubense
Source: Pathogens. 2019 Nov 25;8(4):264. doi: 10.3390/pathogens8040264 (PMC6963284; doi:10.3390/pathogens8040264)
Supplement: Supplementary file 1 [file pathogens-08-00264-s001.pdf]

Supplementary Materials

Table S1. The primers were used for the quantitative real time PCR.

Gene	Primer	Sequence
MaWRKY24	QMaWRKY24-F	GAAGACTTGGAGTTGGGCGG
	QMaWRKY24-R	TCATGAGAAGAAGCTGGAGA
MaATG8f	QMaATG8f-F	ATGGCGAAGAGTTCCTTCAA
	QMaATG8f-R	TTAAACAGATCCAAATGTGT
MaATG8g	QMaATG8g-F	ATGGCGAAGAATTCCTTCAA
	QMaATG8g-R	CTACGCAGACCCAAATGTGT
MaRPS2	QMaRPS2-F	TAGGGATTCCGACGATTTGTTT
	QMaRPS2-R	TAGCGTCATCATTGGCTGGGA
MaATG8f	QChIP-MaATG8f-1F	TCCATCTTGTTATTAGTATC
	QChIP-MaATG8f-1R	AAACAAAAGTCAAAATCC
	QChIP-MaATG8f-2F	GCAGAAGTTGCTTGGATA
	QChIP-MaATG8f-2R	ATTAGTGGAGCTTTAGTT
MaATG8g	QChIP-MaATG8g-1F	TTCGCTGGCAATAACGGACTT
	QChIP-MaATG8g-1R	TTCTTGTGCTCGGCGGTGG
MaACT	QChIP-MaACT-F	AAGGTCAGTCGACTTCGTCTTC
	QChIP-MaACT-R	AGTTACCAAATGTTCGCTCGAT

Table S2. The primers were used for the vector construction.

Gene	Primer	Sequence	Construction
MaWRKY24	MaWRKY24-F	TCCCCCCGGGATGGAGAAAGGCAAAGCAAG	MaWRKY24-pEGAD
	MaWRKY24-R	CGCGGATCCTCATGAGAAGAAGCTGGAGA	
MaWRKY24	MaWRKY24-F	CGCGGATCCATGGAGAAAGGCAAAGCAAG	MaWRKY24-pET28a
	MaWRKY24-R	AAGGAAAAAAGCGGCCGCTGAGAAGAAGCTGGAGAAGT	
MaATG8f	pMaATG8f-F	CGGGGTACCATATTTTGTAGTGTGTAAAT	pMaATG8f-pGreen II 0800-LUC
	pMaATG8f-R	TCCCCCCGGGAAGGATTTGATAAATATAAG	
MaATG8g	pMaATG8g-F	CGGGGTACCCAACCATGCGGAGGTAGTCG	pMaATG8g-pGreen II 0800-LUC
	pMaATG8g-R	TCCCCCCGGGCTCGTCGACCAAGATCGCG	
W-box-mini35S	W-box-mini35S-F	TCCCCCCGGGTTGACCTTGACTTTGACCTTGACTTTGACCTTGACGATATCTCCACTGACGTAAGGG	pW-box-mini35S-pGreen II 0800-LUC
	W-box-mini35S-R	CGCGGATCCCGTGTTCTCTCCAAATGAAATG	
mW-box-mini35S	mW-box-mini35S-F	TCCCCCCGGGTTCTGGTTCTGTTTCTGGTTCTGTTTCTGGTTCTGGATATCTCCACTGACGTAAGGG	pmW-box-mini35S-pGreen II 0800-LUC
	mW-box-mini35S-R	CGCGGATCCCGTGTTCTCTCCAAATGAAATG	

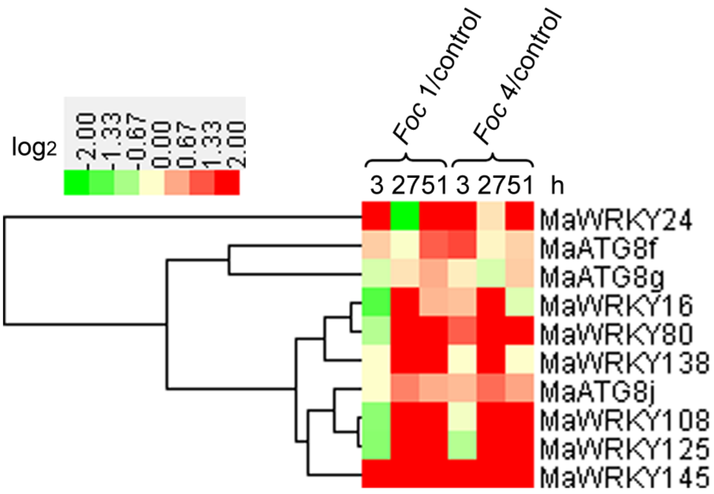

Figure S1. Cluster analysis of correlation between *MaWRKYs* and *MaATG8s* expression. The transcriptomic data were downloaded from Li et al. (2013). For the assay, banana roots were inoculated by *Foc* 1 or *Foc* 4, respectively. The heatmap of gene expression was constructed using CLUSTER program and Java Treeview.
